# Ethnopharmocological study of medicinal plants used for treatment of skin diseases by herbalists in Northwestern region of Algeria

**DOI:** 10.1371/journal.pone.0343714

**Published:** 2026-02-27

**Authors:** Razika Hantour, Yasmina Benabdesslem, Mustapha Diaf, Kadda Hachem, Samia Ghomari

**Affiliations:** 1 Laboratory of Nutrition, Pathology, Agro-Biotechnology, and Health (LAB-NUPABS), Faculty of Natural and Life Sciences, Djillali Liabes University of Sidi Bel Abbès, Sidi Bel Abbès, Algeria; 2 Laboratory of Biotoxicology, Pharmacognosy and Biological Valorization of Plants (LBPVBP), Faculty of Natural and Life Sciences, University of Saida - Dr. Moulay Tahar, Saida, Algeria; 3 Higher Normal School of Saida, Saida, Algeria,; 4 Higher School of Agronomy Mostaganem, Mostaganem, Algeria; University of Milan, ITALY

## Abstract

**Background:**

Skin disease is a major public health concern worldwide, particularly in developing countries. Despite the rich diversity of medicinal plants and strong traditional healing practices in Algeria, the dependence on imported pharmaceuticals remains high. This study documents and analyzes traditional herbal knowledge related to the treatment of skin diseases in Northwestern Algeria (Mascara, Saïda, and Sidi Bel Abbès) with the aim to preserve and scientifically validate this ethnomedicinal heritage.

**Methods:**

A total of 117 herbalists (110 men and seven women) were interviewed using semi-structured questionnaires between September 2023 and July 2024. Data were analyzed using quantitative ethnobotanical indices, including the Relative Frequency of Citations (RFC) and Plant Part Value (PPV), to assess the cultural significance and usage frequency of medicinal species.

**Results:**

A total of 73 plant species belonging to 29 families have been reported for the treatment of various skin ailments. The most abundant families were *Lamiaceae*, *Asteraceae*, *Cucurbitaceae*, *Liliaceae*, *Cupressaceae*, and *Fabaceae*. *Allium sativum* L. and *Thymus vulgaris* L. showed the highest RFC values (0.175 and 0.124, respectively). Leaves were the most frequently used plant parts (PPV = 0.48; 47.6%), followed by fruits (PPV = 0.12; 11.6%), and seeds (PPV = 0.09; 8.9%). Most of the remedies were prepared from dried plants (65.31%), powders (39.9%), and decoctions (22.55%) being the main preparation methods. Topical application (35.2%), poultices (30%), and rinsing (25%) were the most common modes of administration. The most frequently treated skin conditions were eczema, psoriasis, and acne.

**Conclusions:**

This study highlights the remarkable ethnopharmacological knowledge preserved among herbalists in Northwestern Algeria, including women’s traditional roles in medicinal practices. The documentation of these findings not only assists preserve valuable cultural heritage but also provides a foundation for future pharmacological research and the sustainable use of the rich medicinal flora of Algeria.

## Introduction

Skin disease is a major public health concern globally. On May 24, 2025, the seventy eighth World Health Assembly (WHA78) adopted a resolution that recognized skin diseases as a global health priority. This resolution reflects the collective commitment of the World Health Organization (WHO) Member States to strengthen policies, mobilize resources, and improve care for individuals with dermatological disorders [1].

Skin diseases are the fourth leading cause of nonfatal disease burden worldwide and are associated with significant disability, social stigma, psychological distress, and economic expenditures, including substantial out-of-pocket expenses. They are also frequently linked to comorbidities such as depression, cardiovascular disease, and allergies [2].These disorders encompass a broad spectrum of infectious, inflammatory, autoimmune, congenital, chronic, and neoplastic diseases, many of which remain underdiagnosed and undertreated, particularly in low- and middle-income countries [3,4].

Skin and subcutaneous diseases are among the leading causes of morbidity and mortality in the Middle East and North Africa (MENA) region in 2021. The most prevalent conditions include cutaneous mycoses, atopic dermatitis, contact dermatitis, scabies, acne vulgaris, pruritus, urticaria, and cutaneous leishmaniasis [4].

Traditional medicinal plants, used for generations by local populations, represent a key therapeutic resource and major source of novel bioactive compounds [5]. In many developing countries, they constitute the foundation of primary healthcare owing to their accessibility, affordability, and cultural relevance. However, this natural heritage is increasingly threatened by habitat degradation, overexploitation, and climate change, highlighting the need for integrated conservation strategies that combine in situ and ex situ approaches while safeguarding traditional knowledge [6,7].

As of 2025, nearly 80% of the world’s 8.2 billion inhabitants rely on plant based traditional medicine, reflecting both the deep rooted development of indigenous knowledge systems and the limited access to conventional healthcare in resource constrained settings [7,8].

Phylogenetic studies of bioactive plant lineages provide a robust framework for exploring new medicinal resources because plant diversity offers extensive chemical, genetic, and therapeutic potential. Recent advances, including DNA barcoding, AI-assisted phytochemistry, phylogenetic profiling, and geomatic tools, such as GIS and remote sensing, have significantly improved the identification, classification, and spatial mapping of medicinal plant species with high therapeutic value. These technologies enhance traceability, support sustainable resource management, and accelerate the discovery of new bioactive agents, including those effective against skin-related disorders [8–10].

Over the past few decades, numerous ethnobotanical studies have documented medicinal plants used to treat dermatological conditions [11]. In the Maghreb region, several ethnopharmacological surveys have reported the therapeutic uses of plants in Morocco [12,13], Tunisia [14,15], and Algeria, particularly in the Aïn Timouchent and Algiers regions [16,17]. Nevertheless, few studies have specifically focused on the dermatological uses of plants in Western and Northwestern Algeria, despite their remarkable ecological and floristic richness [18].

Women contribute to the preservation and transmission of traditional medicinal knowledge in Algerian communities. However, this study primarily reflects the practices of herbalists in formal and commercial settings in northwestern Algeria, where herbalism is mostly practiced by men. Thus, it is evident that the findings would capture knowledge accessible through these channels, while women’s knowledge, often transmitted in domestic and informal contexts, would remain underrepresented. This highlights the need for studies that focus on these perspectives.

Therefore, the present study aimed to document, quantify, and analyze the medicinal plants used by herbal practitioners for the treatment of dermatological disorders in Northwestern Algeria, with particular attention to practices involving women. Based on a systematic ethnopharmacological approach, this study provides the first detailed ethnodermatological investigation of this region. Our findings fill a scientific gap while contributing to the conservation and sustainable valorization of local medicinal plant biodiversity.

## Materials and methods

### Study area

**Fig 1:**
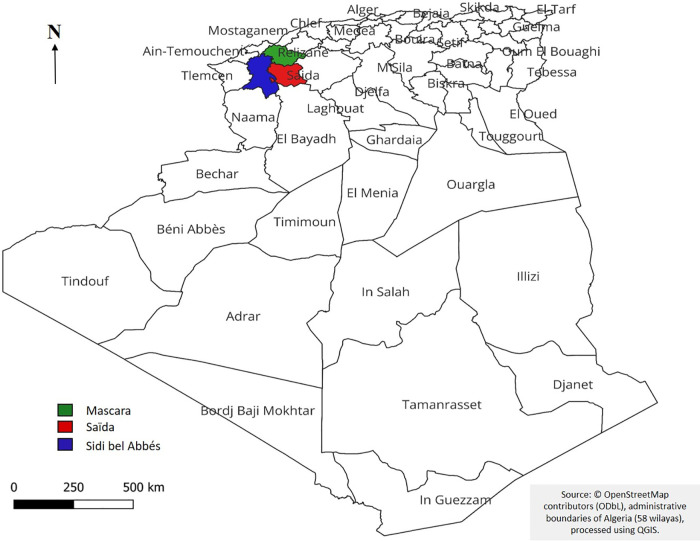
Geographical location of study area (Mascara, Saïda, Sidi Bel Abbès). Base map: © OpenStreetMap contributors. Data available under the Open Database License (ODbL) [19].

The study was conducted in Northwestern Algeria, covering three provinces: Mascara, Saïda, and Sidi Bel Abbès (Fig 1), situated in the western part of the Tell Atlas range.

This region is characterized by diverse topography, including mountain ranges, plains, and steppe zones, which together shape the ecological gradients that influence plant diversity and traditional medicinal practices [20–22].

In Mascara, situated between the Dhaya and Beni Chougrane Mountains, the climate is Mediterranean with a semi-arid tendency, characterized by a mean annual temperature of 17.1 °C, summer peaks reaching 43.5 °C, and an average annual rainfall of approximately 370 mm [20]. The province of Saïda, dominated by the Saïda Mountains, receives 300–350 mm of rainfall annually, with winter minima approximately 2 °C and summer maxima reaching 35 °C [21]. Sidi Bel Abbès, located between the Tellian and High Plateaus regions, has an average annual temperature of 15.7 °C, with minimum averages of 8.3 °C in January and maximum averages of 24.6 °C in August, and annual precipitation of about 442 mm [22]. The vegetation across these provinces consists mainly of natural forests, maquis, and scrublands dominated by Aleppo pine (*Pinus halepensis*) and juniper (*Juniperus oxycedrus*), which are typical of the Oranian Tell. This ecological heterogeneity supports rich floristic diversity and underpins the wide range of medicinal plants traditionally used for dermatological and therapeutic purposes in the region [20–22].

The region of Mascara is located in northwestern Algeria (latitude, 35° 23′ 40 N; longitude, 0° 08′ 23 E; altitude, 492 m). It covers an area of 5135 km². It is limited to the north by Oran and Mostaganem, east by Tiaret and Relizane, west by Sidi Bel Abbès, and south by Saïda [23]. The wilaya of Saïda is located in the northwest of Algeria at coordinates 34° 50′ 00″ N, 0° 09′ 00″ E. It spans an area of 6764 km². Saïda is bordered by the wilaya of Mascara to the north, El-Bayadh to the south, Sidi Bel Abbès to the west, and Tiaret to the east [24]. The wilaya of Sidi Bel Abbès is located in the northwest of Algeria, with coordinates 35° 11′ 38″ N and 0° 38′ 29″ W. It covers an area of 9150.63 km². The region is bordered to the north by Tlemcen and Ain Timouchent, southeast by Mascara, southwest by Saïda, and south by El Bayadh [25].

### Ethnopharmacological survey and data collection

The ethnopharmacological research was undertaken for a period of ten months, spanning from September 2023 to July 2024. The population of the sample comprised 117 herbalists, defined as vendors of medicinal plants in local markets, from the wilayas of Mascara, Saïda, and Sidi Bel Abbès.

Participants were randomly selected from market vendors, which serve as the main distribution points for medicinal plants, ensuring representativeness and minimizing selection bias. The sample size in this study was determined based on data saturation, as interviews continued until no new plant species or significant traditional uses were reported.

Data were collected through semi-structured interviews, supplemented by a predesigned structured questionnaire (https://rb.gy/xfz9p3) composed of approximately 16 items divided into two main sections. The questionnaire was pre-tested with ten vendors, refined based on feedback, and subsequently validated by two experts to ensure clarity, cultural relevance, and content reliability before full deployment.

The first section gathered sociodemographic information of each participant, including age, sex, education level, marital status, and location. The second section focused on traditional dermatological practices, recording details such as the plant’s local name, type (wild, cultivated, or imported), part used, state (fresh or dried), preparation method (decoction, infusion, maceration, powder, oil, etc.), mode of administration (oral, topical, poultice, massage, fumigation), dosage, duration of use, season of collection, association with other substances (e.g., honey, olive oil, water), and therapeutic results (cure, improvement, or relief).

Medicinal plants cited by informants were collected from local markets and natural habitats during field surveys in Mascara, Saïda, and Sidi Bel Abbès. Vernacular names were recorded and cross-checked with standard botanical references (Flora of Algeria; African Plant Database). Taxonomic identification (scientific names and families) was verified by a university botanist to ensure accuracy and reliability of the data.

Interviews were conducted in the local Algerian Arabic dialects to ensure clarity and comprehension. For participants with limited literacy, the interviewers used a simplified and culturally appropriate language to facilitate accurate responses. Before each interview, the participants were fully informed about the study objectives, procedures, voluntary nature of their participation, and the intended use of the collected data for research and publication. All data were anonymized using numerical codes, securely stored in both electronic and paper formats, and accessed only by the research team to ensure participant confidentiality.

Verbal consent was obtained from all participants, documented in writing and signed by the interviewer, and in some cases verified by a second team member to ensure accuracy and adherence to ethical standards. Participation was voluntary, excluding minors, and confidentiality of all responses was guaranteed. The interviews lasted between 15 and 30 min each.

### Data management

All data were entered and analyzed using SPSS version 27 and Microsoft Excel 2010.

### Quantitative ethnopharmacological data analysis

Descriptive statistics were used to summarize participant demographics and frequencies of reported plant species.

Beyond the descriptive analysis, a set of standardized ethnopharmacological indices was applied to quantify the cultural and therapeutic relevance of each plant species. Specifically, the relative frequency of citations (RFC) and plant part values (PPV) were calculated. These indices provided insights into how frequently each species was cited, its relative cultural importance, and the specific ways in which it was used within the studied communities.

### Frequency of citation (FC)

The frequency of citations allowed us to assess the credibility of the information received and the level of knowledge about plants among the surveyed population.

The frequency of citations (FC) of a species corresponds to the number of respondents who mentioned that species [26].

### Relative frequency of citation (RFC)

The RFC index measures how often a plant is identified as a remedy by people providing information and is calculated by dividing the number of informants who mention a particular plant by the total number of informants. Equation 1 defines the RFC in terms of FC and N:


RFC = FC/ N (1)

(0 < RFC < 1)

where FC is the number of informants mentioning the use of the species, and N is the number of informants participating [27].

### Plant part value (PPV)

Plant part value (PPV) was calculated using equation 2:


$$\text{PPV}={\text{RU}}_{Plant\;part}/\text{ RU}$$ (2)

where RU is the number of uses reported for all parts of the plant and RU_Plant part_ is the sum of number of uses reported per part of the plant [27]. The part with the highest PPV was used the most by the respondents.

These indices were compared by province and plant family to identify usage patterns, consensus among informants, and species of greatest cultural and therapeutic importance. High RFC and PPV values indicated cultural significance and perceived efficacy, guiding the selection of species for subsequent pharmacological studies.

## Results

### Demographic characteristics of informants

Of the respondents, 110 were men and seven women. The respondents ranged in age from 20 to 61 years. In terms of representation, at 38.5%, the largest group of respondents was 41–50 years, followed closely by the 31–40 and 51–60 age groups at 25.6% and 15.4%, respectively. The youngest age group (20–30 years) accounted for 9.4% of the total sample. The mean age of the respondents was approximately 44.8 years, with a median age of 44.4 years, and an age range between 20 and 68 years. Most respondents had a secondary school education (41.9%) or intermediate education (22.2%), and 18.8% had university education. Furthermore, 35.9% of the respondents were single, and 64.1% were married. The regional distribution of participants, as shown in Table 1, revealed that rural respondents represented the majority in Mascara (57.5%) and Saïda (56.8%), whereas urban respondents were more prevalent in Sidi Bel Abbès (57.6%).

**Table 1:** Sociodemographic profile of informants.

Parameters	Groups		Numbers	Percentage (%)
**Sex**	Female		7	6
	Male		110	94
**Family situation**	Married		75	64.1
	Single		42	35.9
**Age (years)**	20-30		11	9.4
	31-40		30	25.6
	41-50		45	38.5
	51-60		18	15.4
	61>		13	11.1
	Mean age		44.8 years	
	Median age		44.4 years	
	Age range		48.0 years	
**Education level**	No level		1	0.9
	Primary		19	16.2
	Intermediate		26	22.2
	Secondary		49	41.9
	University		22	18.8
**Province (wilaya)**	Mascara	Total informants	40	34.2
		*Urban*	*19*	*42.5*
		*Rural*	*21*	*57.5*
	Saïda	Total informants	44	37.6
		*Urban*	*19*	*43.2*
		*Rural*	*25*	*56.8*
	Sidi Bel Abbès	Total informants	33	28.2
		*Urban*	*19*	*57.6*
		*Rural*	*14*	*42.4*

### Floristic analysis

This study identified 73 species of medicinal plants from 29 families that were used to treat skin conditions. Of these, Lamiaceae, Asteraceae, Cucurbitaceae, Lilaceae, Cupressacea, and Fabaceae were the most frequently identified families (S1 Appendix). All scientific names are italicized and formatted according to POWO standards [28].

### Parts used

Phytotherapy involves the use of plant parts such as seeds, aerial parts, leaves, flowers, fruits, rhizomes, bulbs, roots, and sometimes the entire plant.

**Fig 2:**
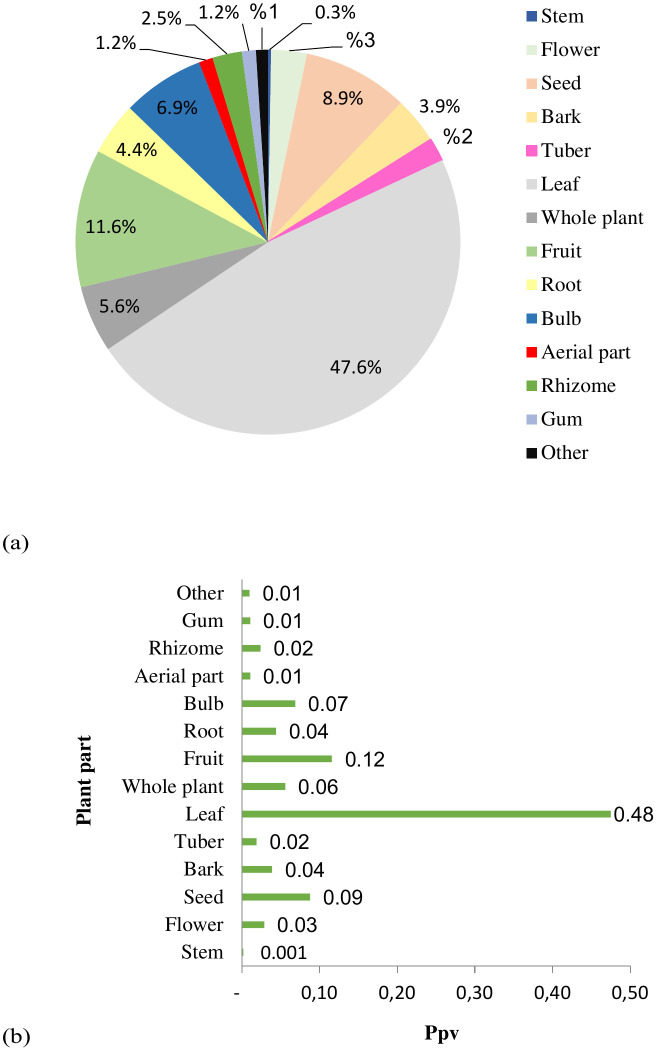
Plant parts used in treatment of dermatological diseases in the study area: (a) Percentage of plant part used; (b) Plant part index for skin disease treatments.

Of all the parts of the plant used for the preparation of dermatological medicines in the survey area, the leaves were by far the most frequently utilized, accounting for nearly half of all citations (PPV = 0.48; 47.6%). Fruits (PPV = 0.12; 11.6%), seeds (PPV = 0.09; 8.9%), and bulbs (PPV = 0.07; 6.9%) were the second most commonly used parts, indicating their moderate ethnomedicinal importance (Fig 2).In contrast, other plant organs, such as roots, bark, and flowers, showed considerably lower PPV values, reflecting their limited contribution to traditional dermatological formulations and suggesting a preferential reliance on the aerial parts owing to their accessibility and ease of processing.

### Species used for dermatological conditions

The relative frequency of citation (RFC) of medicinal plants in the studied area ranged from 0.003 to 0.175, with a mean of 0.035, a median of 0.022, and a standard deviation of 0.035 (Table 2, S1 Appendix), indicating considerable variation in their cultural importance. The two species with the highest RFC were *Allium sativum* L. (RFC = 0.175) and *Thymus vulgaris* L. (RFC = 0.124).

**Table 2:** Most used medicinal plants cited by informants.

Family	Scientific name	RFC
Amaryllidaceae	*Allium sativum* L.	0.175
Lamiaceae	*Thymus vulgaris* L.	0.124
	*Teucrium polium* L.	0.067
	*Lavandula angustifolia* Mill.	0.067
Cucurbitaceae	*Citrullus colocynthis* (L.) Schrad.	0.096
Cupressaceae	*Juniperus oxycedrus* L.	0.067
Anacardiaceae	*Pistacia lentiscus* L.	0.062
Solanaceae	*Hyoscyamus albus* L.	0.062
Cucurbitaceae	*Ecballium elaterium* (L.) A. Rich.	0.062
Ephedraceae	*Ephedra alata* subsp. *alata*	0.056

Plants with high RFC values (> 0.07), such as *Allium sativum* L., *Thymus vulgaris* L., and *Citrullus colocynthis* (L.) Schrad., were the most culturally significant and were frequently cited by the informants, reflecting their strong ethnobotanical relevance and widespread therapeutic applications. Species with moderate RFC values (0.02–0.07), including *Artemisia herba-alba* Asso and *Curcuma longa* L., were moderately known and used, suggesting a balanced yet less dominant role in local traditional medicine.

In contrast, plants with low RFC values (< 0.02), such as *Nigella damascena* L. and *Lepidium sativum* L., were rarely mentioned, indicating limited local utilization or the use of specific, less common treatments. Based on these results, medicinal plants were categorized into three levels of cultural significance, high, medium, and low,–highlighting the heterogeneity of ethnopharmacological knowledge and usage within the study population.

### Plant condition of herbal remedies

**Fig 3:**
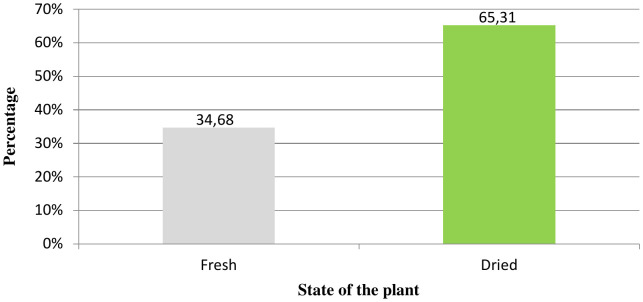
Percentage of fresh and dried plant types.

Many herbalists prefer to use dried herbs over fresh commodities. This was reflected in our results, which revealed that 34.68% of the medicines in the sampled areas were made from fresh parts and 65.31% from dried parts (Fig 3).

### Methods of remedy preparation

Traditionally, ancient preparation methods such as decoction, infusion, powder, raw, and maceration are used to realize the maximum effect of the active principles of a plant.

**Fig 4:**
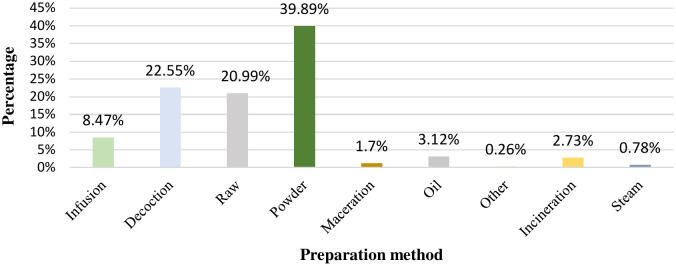
Percentage of different methods of preparation.

Fig 4 illustrates that the most widely reported method of preparation for skin diseases was powder, accounting for 39.90% of responses. This was followed by the decoction at 22.55%, raw at 20.99%, and infusion at 8.47%.

### Modes of administration of conventional treatments

Conventional treatments can be applied using various methods, including oral consumption, topical application, massage, rinsing, and poultices, among others. The choice of the best method depends on the characteristics of the condition to be cured and the vehicle used for the treatment.

**Fig 5:**
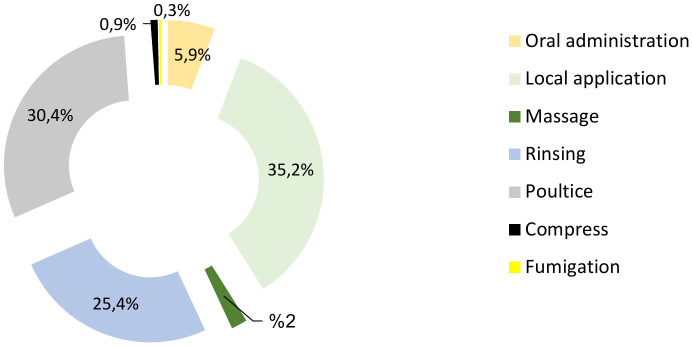
Methods of administering traditional remedies.

The most frequently reported method was local application (Fig 5), which accounted for 35.2% of the cases, followed by poultices and rinsing (30.4% and 25.4%, respectively). The remaining 9% comprised the oral route (5.9%), massage (2%), compression (0.9%), and fumigation (0.3%). The explanation why the percentages do not sum to 100% is that in traditional practices a single species may be applied via multiple routes depending on the condition being treated. Allowing for multiple responses ensures an accurate representation of the diversity and frequency of ethnomedicinal practices.

### Skin conditions treated

This study provides information on the folklore applications of medicinal plants for the treatment of skin diseases in Northwestern Algeria.

**Fig 6:**
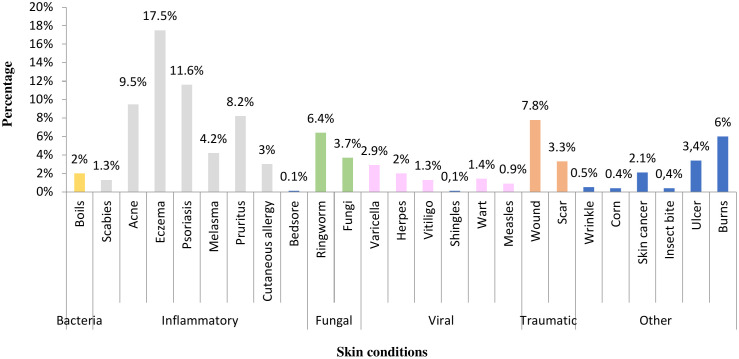
Ailment treated by medicinal plants cited.

One survey showed that inflammatory dermatoses were the most commonly cited disorders. Eczema, psoriasis, and acne were the most frequently cited diseases, with a high percentage of skin diseases cured with local medicinal plants (Fig 6, Table 3).

**Table 3:** Medicinal plants used for skin conditions.

Plant	Treated skin conditions
*Allium sativum* L.	**Bacterial:** Boils **Inflammatory:** Acne, Eczema, Psoriasis,Pruritus **Fungal:** Ringworm, Onychomycosis, Fungal infections **Viral:** Herpes, Wart
*Thymus vulgaris* L.	**Bacterial:** Scabies, Boils **Inflammatory:** Acne, Eczema, Psoriasis, Pruritus **Fungal:** Ringworm **Viral:** Varicella, Measles **Other:** Skin cancer
*Teucrium polium* L.	**Inflammatory:** Acne, Eczema **Traumatic:** Wound **Other:** Ulcer, Skin cancer
*Lavandula angustifolia* Mill.	**Inflammatory:** Acne, Eczema, Psoriasis, Pruritus **Fungal:** Ringworm, Cutaneous mycoses, Fungal infections
*Citrullus colocynthis* (L.) Schrad.	**Inflammatory:** Acne, Eczema, Psoriasis **Fungal:** Ringworm, Onychomycosis, Fungal infections **Other:** Wrinkle
*Juniperus oxycedrus* L.	**Bacterial:** Scabies **Inflammatory:** Acne, Eczema, Psoriasis, Pruritus **Fungal:** Ringworm, Fungal infections **Traumatic:** Wound, Bedsore
*Pistacia lentiscus* L.	**Inflammatory:** Eczema, Psoriasis, Pruritus **Fungal:** Ringworm, Cutaneous mycoses **Traumatic:** Wound **Other:** Burns
*Hyoscyamus albus* L.	**Inflammatory:** Eczema, Psoriasis, Pruritus **Fungal:** Ringworm **Viral:** Varicella, Measles, Herpes **Other:** Burns, Skin cancer
*Ecballium elaterium* (L.) A. Rich.	**Inflammatory:** Eczema, Psoriasis **Fungal:** Fungal infections **Other:** Skin cancer
*Ephedra alata* subsp. *alata*	**Inflammatory:** Acne, Eczema **Viral:** Varicella, Measles **Traumatic:** Wound **Other:** Burns, Ulcer, Skin cancer

## Discussion

Most of the interviewed herbal practitioners were men (94%), reflecting the gendered division of labor in Northwestern Algeria, where men are primarily responsible for plant collection, trade, and public healthcare, whereas women mainly preserve and transmit ethnopharmacological knowledge within families. The roles of different sexes influence the structure and continuity of traditional medicinal knowledge in a region.

The results obtained by Djarmouni et al. [29] indicated that 97% of herbalists in the Sétif region were men, and only 3% were women, All herbalists assessed in the Saïda region were men [30], indicating that sales of medicinal plants and phytotherapy practices remain male-dominated. Moreover, community customs have long inhibited women from working outside their homes. However, their participation in herbalism has gained momentum, as shown by the emergence of groups and shops specializing in them [31].

According to a study conducted in Morocco using similar criteria, 49% of herbalists, belonging to the adult group (31 years and older), were married [32]. With respect to age, our results align with two national studies which showed that 43.75% and 31% of herbalists, respectively, were in the 40–50 year age group, the dominant age group in herbal medicine practice [29,33]. Older people are perceived as knowledgeable about medicinal plants and their uses because of their vast experience and the transmission of traditional knowledge [34].

Regarding the educational background of herbalists, most of them usually completed secondary school. According to national studies conducted by Djahafi et al. [33], Djarmouni et al. [29], and Benabdesslem [35], 52.38%, 42%, and 35% of herbalists, respectively, completed secondary school [35]. Other studies have cited lower formal education levels working quite well in indigenous plant knowledge in traditional medicine. This has been documented in both Algeria [36] and other countries [37–39].

In the past, herbalists and their practices suffered because war, poverty, and other socioeconomic conditions resulted in inadequate education. Today, people actively seek information because they are inquisitive and knowledgeable. This allows one to keep in touch with the most recent advancements related to plants and ailments, thereby enhancing the sale of these plants and fine-tuning their skills through elaborate research and a better understanding of medicinal plants. Nevertheless, the exceedingly high illiteracy rate continues to be an overwhelming obstacle in the development of traditional medicine [31].

According to earlier ethnobotanical surveys conducted in Morocco, the Lamiaceae family is most often used to treat skin conditions, as reported by Hilal et al. [40], Mouchane et al. [41], and other earlier findings.

Tsioutsiou et al. [42] indicated that Asteraceae and Lamiaceae were two of the most represented families of medicinal plants in East Mediterranean and South Balkan regions. In Morocco, however, Solanaceae wasthe most commonly used family for the treatment and prevention of skin-related issues, and the Apocynaceae family predominated in Senegal, consistent with previous studies [12,43,44].

The Lamiaceae family, one of the most important herbal families, includes a wide variety of plants with biological and therapeutic applications. The most well known members of this family are a variety of aromatic species, such as thyme, mint, oregano, basil, sage, savory, rosemary, self-healing, hyssop, lemon balm, and others with limited use [45,46]. These plants contain varied secondary metabolites, which include polyphenols, aromatic alcohols, volatile terpenes, diterpenoids, triterpenoids, flavonoids, coumarins, tannins, and other substances that have antimicrobial and antioxidant properties in some of them [46–49]. Therefore, members of the Lamiaceae family are highly regarded as potent antioxidants and have a wide range of industrial applications in aromatherapy, cosmetics, and medicine [50]. Plants of this family are used in the traditional African pharmacopoeia for the treatment of various skin ailments and for their antibacterial and wound-healing properties [51–55].

Plants of the Asteraceae family are recognized for their bioactive compounds that have various therapeutic properties. It has been suggested that further research maylead to the development of new treatments for diseases such as ulcers [56].

As for the organs, the plant part mentioned the most was the leaves. There is variability in the concentration of active compounds in each plant organ and, consequently, in the proportion of plant parts employed [43].Leaves are most often selected because they are easy to find, medication preparation is simple, and they contain secondary metabolites that carry out photosynthesis [43].

Studies by Benabdesslem [35], Ouadeh et al. [57], Bentabet et al. [16], and Meddour et al. [58] in Algeria demonstrated the use of leaves with rates of 69%, 22%, 28%, and 27.9%, respectively. Similar results were reported by Diatta et al. [43] and Dramane et al. [34] with rates of 40% and 46%, respectively, in Senegal. Studies in Morocco [12,40,41,59–61] also supported leaves as the most common plant parts used, with proportions of 39.33%, 28%, 41%, 32%, 38%, and 53.71%, respectively, whereas Ralte and Singh [62] reported a proportion of 30.39% in India.

A related study indicated that the highest RFC value was found for garlic (*Allium sativum* L.). Moreover, according to Bentabet et al. [16], frequent uses among the plant treatments for dermatological disorders were fenugreek (*Trigonella foenum-graecum* L.) and thyme (*Thymus vulgaris* L.). Consistent with these findings, our survey revealed that *Thymus vulgaris* and *Allium sativum* were the most frequently cited species, highlighting their significance in local ethnomedicine and their pharmacological relevance. Their widespread use suggests that these plants are effective and easily accessible for managing skin and infectious conditions.

*Allium sativum* L., commonly known as garlic, contains thiosulfinates (alliins), ajoenes, sulfides, vitamins, flavonoids, minerals, and phenolic compounds. Itexhibits antioxidant, antibiotic, antifungal, antibacterial, antiirritant, anti-inflammatory, antiseptic, and antiparasitic properties. Ajoenes display activity against *Candida albicans*, whereas allicin shows bactericidal and bacteriostatic effects, inhibiting the development of staphylococci, fungi, and yeasts. Pharmacologically, it is applied topically to treat arthritis, corns, warts, ulcers, burns, dermatophyte infections, and various skin conditions [63,64]. *Thymus vulgaris* L., or thyme, is another plant known for its therapeutic properties and is rich in essential oils (thymol and carvacrol), flavonoids, and phenolic compounds. They have antioxidant, antimicrobial, antibacterial, antifungal, and anti-inflammatory properties. The extracts showed inhibitory activity against herpes simplex virus types 1 (HSV-1) and 2 (HSV-2). It is used to treat skin problems, acne, dermatitis, inflammatory skin conditions, insect bites, and alopecia [65,66].

In this study, most of the remedies were prepared using the dried parts. A study conducted in Northeastern Morocco [67] reported that 91% of plant parts commonly used were dried for use.

Since green plants may not always be available, drying is necessary for prolonged storage. This guarantees plant availability throughout the year, including during the off-peak seasons. Attention is focused on the drying process to ensure maximum retention of the active ingredients of the plants.

Hilal et al. [40] showed that powder preparation is the most common method, with a prevalence of 58.66% in Morocco’s central plateau. Ajjoun et al. [12] found that powder and decoction were the most commonly employed traditional methods for treating skin diseases in Morocco. Dramane et al. [34] identified these methods as commonly used in the treatment of fungal diseases in Côte d’Ivoire.

Powdering medicinal plants is an effective method for preserving active constituents, such as flavonoids, tannins, and terpenes, which can be degraded by heat treatment [68]. Plant material, either in the form of specific parts or whole plants, is harvested at the optimal time to obtain high yields, and then washed and dried, primarily at low temperatures, to preserve the activity of thermolabile compounds. Upon drying, the material is powdered, which increases its surface area and enhances the extraction of bioactive compounds from the plant matrix [69]. In contrast, decoctions are aqueous preparations used to extract the active components of medicinal plants. However, this method lacks selectivity, particularly for compounds that are insoluble in water [70].

The powder form retains the anti-inflammatory, antioxidant, and healing properties of such compounds, a factor that makes this form particularly well suited for applications involving the treatment of skin diseases that require a high concentration of natural bioactive constituents. Therefore, the powder remains the most effective and convenient form for such applications.

The majority of the prepared remedies are applicable for topical applications and poultices. These findings concur with those of Orsot et al. [71], who reported that local application is the most common method, particularly for skin infections, where medications are applied topically to the affected site. Dramane et al. [34] also observed that local application was the most commonly used method for treating fungal infections in Africa. Bentabet et al. [16] reported that washing is the most frequent treatment practice for dermatoses in the Ain Temouchent region.

Topical therapy is an effective approach for treating skin illnesses [72]. By penetrating the stratum corneum, the process allows therapeutic agents to directly access the diseased skin sites [73]. It is designed to deliver localized bactericidal, antifungal [74,75], and healing actions, particularly to superficial dermatoses. The incorporation of active ingredients is greater if the skin is damaged or softened; or it can be enhanced by adding adjuvants such as olive oil or honey, which permit greater penetration.

Bentabet et al. [16] reported that at the national level in Wilaya of Ain Temouchent, acne accounts for 20%, followed by skin lightening and eczema at 10% each. Similarly, an ethnobotanical survey conducted by Ostor et al. [56] in South Africa identified diaper dermatitis as the most prevalent skin condition, with a rate of 12.81%. However, studies by El Azzouzi et al. [44] demonstrated skin cancer to be the most commonly reported skin disease, whereas, Chaachouay et al. [39] reported that dermal wounds were the most common, followed by eczema and herpes wounds.

Diets and oxidative stress are implicated in skin barrier damage, microbiome dysbiosis, and persistent inflammation [76,77]. Sugar-dense, fat-dense, and processed diets correlate more directly with the onset of certain autoimmune conditions, such as psoriasis, than obesity [78]. Furthermore, data show that oxidative stress is one of the key causes of malignant skin tumor formation [77]. However, future studies are required to further substantiate the scientific validity of these findings.

Bioassay-guided studies are recommended to identify and quantify bioactive compounds, whereas toxicological assessments are essential for potentially harmful species such as *Nerium oleander* L., *Atractylis gummifera* L., and *Peganum harmala* L. In addition, developing policy frameworks to integrate traditional dermatological care into primary healthcare systems may ensure the safe and effective use of medicinal plants, thereby bridging traditional knowledge with modern medical practices.

## Limitations

This study has some limitations that should be considered when interpreting the findings. The sample was predominantly male (94%), which may introduce selection bias and limit the generalizability of the results to a broader population, including female herbal practitioners with different ethnopharmacological knowledge. Participants relied on memory to report plant use, preparation methods, and treatment regimens, which may lead to recall bias; precise information on dosages and treatment schedules was often lacking, limiting the reproducibility and comparability of the findings. Additionally, the study did not include voucher specimens deposited in recognized herbaria, which may affect the scientific validation of species identification and hinder reproducibility. Future research should ensure the collection and deposition of voucher specimens to allow accurate identification and facilitate comparison with other ethnopharmacological studies. It is also important to acknowledge the potential toxicity of certain medicinal plants traditionally used to treat dermatological disorders. It is well documented that if improperly used, species such as *Nerium oleander* L., *Atractylis gummifera* L., and *Peganum harmala* L. exhibit toxic effects, including cardiotoxicity, neurotoxicity, and gastrointestinal complications. To enhance the scientific validity and ensure safe practice, bioassay-guided studies to identify and quantify bioactive compounds, along with toxicological assessments of potentially harmful species, are recommended. Furthermore, the development of regulatory frameworks and risk communication programs may facilitate the integration of traditional dermatological care into primary healthcare systems, ensuring the safe and effective use of medicinal plants, while bridging traditional knowledge with modern medical practice.

## Conclusions

This study is the first ethnopharmacological investigation conducted in Northwestern Algeria, specifically in the Mascara, Sidi Bel Abbès, and Saïda areas, which revealed the richness of conventional ethnomedicinal heritage of these regions. Conventional herbalists use medicinal plants to treat various skin conditions such as acne, eczema, wounds, and psoriasis, maintaining their inherited knowledge and firmly holding on to their heritage. The methods used in this study such as the frequency of citation (FC), relative frequency of citation (RFC), and part plant value (PPV), may prove valuable for the future phytomedicinal and pharma research. Additionally, our findings highlight the utilization of medicinal plants in this region, and the conservation of plant diversity and the traditional technique. Overall, this study not only documents traditional practices, but also serves as a valuable resource for bridging ethnopharmacological knowledge with modern scientific research, supporting both pharmacological exploration and the preservation of cultural and biological heritage.

## Supporting information

S1 Appendix Medicinal Plant Species Identified in the Study. (DOCX)
