# Supplementary material for: Ethnopharmocological study of medicinal plants used for treatment of skin diseases by herbalists in Northwestern region of Algeria
Source: PLoS One. 2026 Feb 27;21(2):e0343714. doi: 10.1371/journal.pone.0343714 (PMC12948081; doi:10.1371/journal.pone.0343714)
Supplement: S1 Appendix — (DOCX) [file pone.0343714.s001.docx]

**S1 Appendix**. **Medicinal Plant Species Identified in the Study.**

| **Family** | **Number of citation** | **Scientific name** | **Part used** | **Preparation** | **FC** | **RFC** |
| --- | --- | --- | --- | --- | --- | --- |
| **Renonculaceae** | 18 | *Nigella damascena* L. | Seeds | Powder | 1 | 0.005 |
|  |  | Nigella sativa L. | Seeds, Leaves | Powder | 7 | 0.039 |
|  |  | Ranunculus bullatus L. | Leaves | Decoction | 1 | 0.005 |
| **Lamiaceae** | 151 | *Ajuga iva* (L.) Schreb. | Whole plant | Powder | 1 | 0.005 |
|  |  | Lavandula angustifolia Mill. | Arial part, Flower, Leaves, Bark | Infusion, Decoction, Powder | 12 | 0.067 |
|  |  | Marrubium vulgare L. | Leaves | Infusion, Decoction, Powder | 6 | 0.033 |
|  |  | Mentha pulegium L. | Leaves, Whole plant | Infusion, Decoction | 3 | 0.016 |
|  |  | Ocimum basilicum L. | Leaves, Seeds | Infusion, Decoction, Powder | 5 | 0.028 |
|  |  | Origanum majorana L. | Leaves | Infusion, Powder | 2 | 0.011 |
|  |  | Rosmarinus officinalis L. | Leaves | Infusion, Decoction | 9 | 0.050 |
|  |  | Salvia officinalis L. | Leaves, Whole plant | Infusion, Decoction | 9 | 0.050 |
|  |  | Teucrium polium L. | Leaves, Flowers | Powder | 12 | 0.067 |
|  |  | Thymus vulgaris L. | Arial part, Seeds, Leaves, Whole plant | Infusion, Decoction | 22 | 0.124 |
| **Lythraceae** | 8 | Lawsonia inermis L. | Leaves | Powder | 5 | 0.028 |
| **Apocynaceae** | 13 | Nerium oleander L. | Leaves, Flowers, Whole plant | Decoction, Powder, Maceration | 6 | 0.033 |
| **Amaranthaceae** | 25 | Arthrophytum scoparium (Pomel) Iljin | Leaves, Flowers, Whole plant, Arial part | Infusion, Decoction, Powder, Maceration | 9 | 0.050 |
| **Asteraceae** | 83 | Anacyclus pyrethrum (L.) Lag. | Roots | Powder | 1 | 0.005 |
|  |  | Anacyclus valentinus L. | Leaves | Infusion | 1 | 0.005 |
|  |  | Artemisia herba-alba Asso | Leaves, Whole plant, Roots | Decoction, Powder | 7 | 0.039 |
|  |  | Atractylis gummifera L. | Leaves, Roots | Infusion, Powder | 4 | 0.022 |
|  |  | Carthamus caeruleus L. | Leaves, Roots | Powder | 2 | 0.011 |
|  |  | Dittrichia viscosa (L.) Greuter | Leaves | Infusion, Decoction, Powder | 7 | 0.003 |
|  |  | Matricaria pubescens (Desf.) Schultz Bip. | Flowers, Leaves | Infusion, Decoction, Powder | 5 | 0.028 |
|  |  | Saussurea costus (Falc.) Lipsch. | Roots | Decoction, Powder | 4 | 0.022 |
|  |  | Silybum marianum (L.) Gaertn. | Leaves | Infusion | 1 | 0.005 |
| **Cucurbutaceae** | 59 | Citrullus colocynthis (L.) Schrad. | Fruit | Raw | 17 | 0.096 |
|  |  | Cucumis sativus L. | Fruit | Raw | 2 | 0.011 |
|  |  | Ecballium elaterium (L.) A. Rich. | Leaves, Fruit, Seeds | Decoction, Raw, Other | 11 | 0.062 |
| **Liliaceae** | 59 | Allium sativum L. | Bulb | Raw | 31 | 0.175 |
|  |  | Allium cepa L. | Fruit, Stem | Raw | 2 | 0.011 |
|  |  | Asphodelus microcarpus Viv. | Tuber | Powder | 1 | 0.005 |
| **Apiaceae** | 30 | Angelica sinensis (Oliv.) Diels | Seeds, Leaves | Raw, Powder | 4 | 0.022 |
|  |  | Bunium bulbocastanum L. | Tuber | Powder | 3 | 0.016 |
|  |  | Bunium mauritanicum Batt. | Tuber | Powder | 1 | 0.005 |
|  |  | Dorema ammoniacum D. Don | Other | Raw | 3 | 0.016 |
|  |  | Foeniculum vulgare Mill. | Seeds | Decoction | 1 | 0.005 |
| **Ephedraceae** | 19 | Ephedra alata subsp. alata | Whole plant, Leaves, Roots | Infusion, Decoction, Powder | 10 | 0.056 |
| **Fabaceae** | 34 | Glycyrrhiza glabra L. | Roots | Decoction, Powder, Oil | 4 | 0.022 |
|  |  | Senna alexandrina Mill. | Leaves | Powder | 1 | 0.005 |
|  |  | Trigonella foenum-graecum L. | Seeds, Leaves | Infusion, Powder | 9 | 0.050 |
| **Amaranthaceae** | 9 | Atriplex halimus L. | Leaves, Whole plant | Infusion, Decoction, Powder | 3 | 0.016 |
|  |  | Celosia argentea L. | Leaves | Powder | 1 | 0.005 |
| **Asphodelaceae** | 25 | Aloe vera (L.) Burm. f. | Leaves | Raw | 9 | 0.050 |
| **Rubiaceae** | 7 | Rubia tinctorum L. | Roots | Powder, Maceration | 2 | 0.011 |
| **Berberidaceae** | 15 | Berberis vulgaris L. | Leaves, Whole plant, Roots | Infusion, Decoction, Powder | 3 | 0.016 |
| **Pinaceae** | 11 | Pinus halepensis Mill. | Bark | Powder, Oil | 6 | 0.033 |
| **Zygophyllaceae** | 29 | Fagonia glutinosa Delile | Leaves, Seeds, Whole plant, Arial part | Infusion, Decoction, Powder | 10 | 0.056 |
|  |  | Peganum harmala L. | Seeds, Leaves | Powder | 3 | 0.016 |
|  |  | Zygophyllum album L. | Fruit | Powder | 2 | 0.011 |
| **Linaceae** | 8 | Linum usitatissimum L. | Seeds | Decoction | 2 | 0.011 |
| **Plantaginaceae** | 8 | Globularia alypum L. | Leaves | Decoction, Powder | 3 | 0.016 |
|  |  | Plantago lanceolata L. | Leaves | Raw | 1 | 0.005 |
| **Cupressaceae** | 40 | Tetraclinis articulata (Vahl) Mast. | Seeds, Fruit | Powder | 2 | 0.011 |
|  |  | Juniperus oxycedrus L. | Bark, Leaves, Fruit | Decoction, Powder, Oil | 12 | 0.067 |
| **Solanaceae** | 29 | Hyoscyamus albus L. | Flowers, Leaves, Whole plant | Infusion, Decoction, Raw, Powder | 11 | 0.062 |
| **Urticaceae** | 4 | Urtica dioica L. | Leaves, Whole plant | Decoction, Powder | 3 | 0.016 |
| **Oleaceae** | 3 | Olea europaea L. | Leaves, Fruit | Powder, Oil | 2 | 0.011 |
| **Rosaceae** | 5 | Rosa damascena Mill. | Flowers | Powder | 2 | 0.011 |
|  |  | Potentilla supina L. | Whole plant | Decoction | 1 | 0.005 |
| **Anacardiaceae** | 19 | Pistacia lentiscus L. | Leaves, Fruit | Powder, Oil | 11 | 0.062 |
| **Zingiberaceae** | 21 | Elettaria cardamomum (L.) Maton | Seeds | Powder | 1 | 0.005 |
|  |  | Curcuma longa L. | Rhizome | Powder | 7 | 0.039 |
| **Burseraceae** | 9 | Boswellia sacra Flueck. | Gum | Infusion, Oil | 4 | 0.022 |
| **Poaceae** | 4 | Avena sativa L. | Seeds | Powder | 2 | 0.011 |
| **Thymelaeaceae** | 5 | Daphne gnidium L. | Leaves, Other | Raw, Powder | 3 | 0.016 |
| **Vitaceae** | 1 | Vitis vinifera L. | Leaves | Steam | 1 | 0.005 |
| **Moraceae** | 1 | Ficus carica L. | Other | Raw | 1 | 0.005 |
| **Rhamnaceae** | 3 | Ziziphus spina-christi (L.) Desf. | Leaves | Maceration | 1 | 0.005 |
|  |  | Rhamnus alaternus L. | Leaves | Powder | 1 | 0.005 |
| **Rutaceae** | 4 | Citrus limon (L.) Osbeck | Fruit | Raw | 1 | 0.005 |
|  |  | Ruta graveolens L. | Arial part | Decoction | 1 | 0.005 |
| **Brassicaceae** | 1 | Lepidium sativum L. | Seeds | Powder | 1 | 0.005 |
| **Malvaceae** | 2 | Hibiscus sabdariffa L. | Leaves | Powder | 1 | 0.005 |
| **Euphorbiaceae** | 1 | Ricinus communis L. | seeds | Oil | 1 | 0.005 |
| **Caryophyllaceae** | 1 | Corrigiola telephiifolia Pourr. | leaves | Powder | 1 | 0.005 |
| **Droséraceae** | 3 | Drosera aliciae Raym.-Hamet | Leaves | Infusion, Decoction, Powder | 2 | 0.011 |
